# Segment specific loss of NFAT5 function in the kidneys is sufficient to induce a global kidney injury like phenotype

**DOI:** 10.1096/fj.202402497R

**Published:** 2025-01-28

**Authors:** Kristina Engel, Vera Anna Kulow, Dmitry Chernyakov, Edith Willscher, Michael Fähling, Bayram Edemir

**Affiliations:** ^1^ Department of Medicine, Hematology and Oncology Martin Luther University Halle‐Wittenberg Halle (Saale) Germany; ^2^ Institute of Translational Physiology Charité – Universitätsmedizin Berlin, Corporate Member of Freie Universität Berlin and Humboldt‐Universität zu Berlin Berlin Germany; ^3^ Institute for Physiology and Pathophysiology, Zentrum für Biomedizinische Ausbildung und Forschung (ZBAF) Witten/Herdecke University Witten Germany

**Keywords:** collecting duct, cytokine signaling, kidney cortex, kidney fibrosis, kidney injury, kidney inner medulla, NFAT5

## Abstract

Nuclear factor of activated T‐cells 5 (NFAT5) is a transcription factor known for its role in osmotic stress adaptation in the renal inner medulla, due to the osmotic gradient that is generated between the renal cortex and renal inner medulla. However, its broader implications in kidney injury and chronic kidney disease (CKD) are less understood. Here we used two different Cre deleter mice (Ksp1.3‐Cre and Aqp2‐Cre) to generate tubule segment and even cell type‐specific NFAT5‐deficient mice and performed extensive gene expression profiling. In both *Nfat5* knockout models, we observed massive changes in gene expression pattern, with heightened inflammatory responses and renal injury, culminating in renal fibrosis. Interestingly, inflammatory responses were much more prominent in the Aqp2Cre^+/−^Nfat5^fl/fl^ mice that lack NFAT5 only in the collecting duct. By analyzing gene expression in the medullary and cortical regions of the kidney separately, we confirmed that the loss of NFAT5 results in kidney injury that extends beyond hypertonic areas. Renal injury correlates with the expression level of genes involved in inflammatory response, injury severity, and cytokine signaling. Thus, NFAT5 is essential not only for adapting to osmotic stress but also for its loss of function, which induces activation of inflammatory response and cytokine signaling that might affect regions with functional NFAT5 expression.

## Graphical abstract

We investigated the consequences of NFAT5 knockout in two mouse models targeting the distal nephron: from the thick ascending limb (TAL) to the collecting duct (CD) and specifically in CD principal cells. Congenital NFAT5 loss induces inflammation, kidney fibrosis, immune cell invasion, and proximal tubular injury. Interestingly, inflammation and associated injury are observed in the cortex despite NFAT5 deficiency being restricted to distal nephron segments or CD cells in the medulla, suggesting a potential feedback mechanism linking distal and proximal nephron segments.

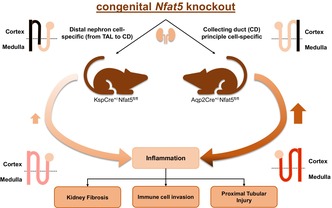

**Abbreviations**
Aqp2: aquaporin‐2
BGT1: sodium coupled betaine transporter
CD: collecting duct
CNT: connecting tubule
Cre: cre recombinase
CTX: cortex
DCT: distal convoluted tubules
F4/80: adhesion G protein‐coupled receptor E1/ADGRE1
FPKM: fragments per kilo base of transcript per million mapped fragments
GO: gene ontology
Havcr1: hepatitis A virus cellular receptor 1
IM: inner medulla
KEGG: Kyoto Encyclopedia of Genes and Genomes
KIM‐1: kidney injury molecule‐1
KO: knockout
Ksp: kidney‐specific
Lcn2: lipocalin‐2
Ly‐6G: lymphocyte antigen 6G
NFAT5: nuclear factor of activated T‐cells 5 (TonEBP)
NGAL: neutrophil gelatinase‐associated lipocalin
NGS: next generation sequencing
NKCC2: Na^+^‐K^+^‐2Cl^−^ co‐transporter/SLC12A1
PC: principal cell
PT: proximal tubule
RNA‐seq: next generation RNA sequencing
TAL: thick ascending limb
TNF: tumor necrose factor
TonEBP: tonicity‐responsive enhancer‐binding factor (NFAT5)
TSS: transcription start sites

## 1 INTRODUCTION

Urine concentration in the mammalian kidney relies on the creation of an interstitial osmotic gradient that promotes water reabsorption from the collecting duct (CD).^1^ This gradient is established through the active transepithelial reabsorption of Na^+^ and Cl^−^ in the outer medulla^1^, ^2^, ^3^, ^4^ and urea in the inner medulla (IM).^5^ As a result, urine osmolality can reach up to 1200 mosmol/kg in humans and approximately 4000 mosmol/kg in mice,^6^ mirroring the osmolality of the inner medullary interstitium.^7^ This creates a hypertonic environment in the medulla, which can be detrimental to cellular functions by causing DNA breaks, hindering DNA repair and proliferation, and potentially leading to cell death.^8^ Despite this, renal medullary cells have evolved adaptive mechanisms to sustain their function. They synthesize osmotically active molecules such as taurine, myo‐inositol, betaine, and sorbitol to balance the ionic strength of the intracellular fluid.^8^

The key factor providing protection under hypertonic conditions is the nuclear factor of activated T‐cells 5 (NFAT5). NFAT5 is activated by stress‐activated kinases, prompting its movement into the nucleus and the initiation of an osmoprotective gene expression program. Its target genes include the heat shock protein *HSP70*,^8^, ^9^, ^10^ various urea transporters, and the water channel aquaporin‐2 (*Aqp2*).^11^, ^12^, ^13^ Global deletion of NFAT5 in mice results almost in embryonic lethality,^14^ causing severe atrophy and cell loss in the renal medulla due to inadequate expression of osmo‐protective genes such as aldose reductase, *BGT1* (*alias SLC6A12*, solute carrier family 6 member 12) and SMIT (*alias SLC5A3*, solute carrier family 5 member 3).^14^ Additionally, NFAT5 deficiency leads to cell cycle arrest by disrupting the expression of G1, S, and G2 cyclins, as well as aurora B kinase. Overexpression of a dominant‐negative NFAT5 in epithelial cells of the thick ascending limb (TAL), distal convoluted tubules (DCT), and CD reduces AQP2 expression and impairs urine concentration.^15^

A recent study using a CD principal cell (PC)‐specific *Nfat5* knockout (*Nfat5*‐KO) mouse revealed that NFAT5 loss is linked to a diabetes insipidus‐like phenotype and significant changes in gene expression.^16^, ^17^ Further, in a Nfat5‐Pax8‐rtTA/LC1 knockout mouse model, Ono et al. found that the loss of NFAT5 deregulated over 2000 transcription start sites (TSS).^18^ Notably, they observed that *Nfat5*‐KO significantly worsened fibrosis in the renal medulla, along with heightened immune response activation following unilateral ureteral obstruction. They concluded that NFAT5 might play critical pathophysiological roles in renal fibrosis by modulating innate and adaptive immune responses. Gene expression analysis in PC‐specific *Nfat5*‐KO mice further indicated that NFAT5 loss is sufficient to induce a kidney injury‐like phenotype.^16^ Therefore, NFAT5 seems to be essential not only for initiating an osmo‐protective response but also for immune system activation. The role of NFAT5 in nephron segments beyond the CD, however, remains unclear.

To explore the impact of spatial *Nfat5*‐KO, we compared transcriptomic data from two different *Nfat5*‐KO models and distinguished between cortex and inner medulla. The first transgenic model involved a knockout driven by the *Aqp2* promoter, causing *Nfat5*‐KO in the principal cells of the CD. The second model used a knockout driven by the Ksp‐promoter, leading to *Nfat5*‐KO in the distal nephron starting in the TAL up to the CD. We hypothesized that gene expression differences would reveal distinct NFAT5 functions. Indeed, NFAT5 loss resulted in a more severe fibrotic phenotype in the Aqp2‐driven *Nfat5*‐KO, which correlated with cytokine signaling in cortical and medullary areas.

## 2 MATERIALS AND METHODS

### 2.1 Sex as a biological variable

Male and female mice were involved in this study. Sex was not considered as a biological variable.

### 2.2 Mouse strains

To generate mice (strain C57BL/6N, RRID:MGI:2159965) that are deficient in NFAT5 in the principal cell (PC) of the collecting duct (CD), floxed *NFAT5* mice (Nfat5^fl/fl^) were crossed with Aqp2‐Cre^+/−^ mice expressing CRE recombinase under the control of the *Aqp2* gene promoter.^17^ The insertion of the Cre recombinase disrupts one allele of the *Aqp2* gene. Therefore, we used only Aqp2Cre^+/−^Nfat5^fl/fl^ mice with Aqp2‐Cre^+/−^ mice as controls. The Nfat5^fl/fl^ mice were kindly provided by Prof. C. Küper.^19^ The Aqp2‐Cre^+/−^ deleted mice were kindly provided by Dr. J. Hadchouel.^20^ To generate distal tubule and CD‐specific deficient mice (KspCre^+/−^Nfat5^fl/fl^) we used Nfat5^fl/fl^ mice and crossed them with KspCre^+/−^ (Ksp1.3‐Cre) mice carrying a 1329 bp of the Ksp‐cadherin (kidney‐specific Cadherin 16) 5′ flanking region linked to the Cre recombinase gene. The Ksp1.3‐Cre mice were obtained from The Jackson laboratories (Bar Harbor, ME, USA,^21^). Mice genotyping was conducted as previously described.^16^, ^20^, ^21^ The mice were bred in the research laboratory's own animal facility of the University Halle‐Wittenberg. The animals were euthanized by CO_2_ inhalation in accordance with institutional guidelines, followed by immediate harvesting of the kidneys for further analysis. For the next generation sequencing, the cortex and the inner medulla tissue of the same kidney from 21‐week‐old mice were used. In total, kidneys from 31  adult mice were used: *n* = 6 from the Aqp2Cre^+/−^Nfat5^fl/fl^ group (3 male/3 female), *n* = 11 from the Aqp2‐Cre^+/−^ group (4 male/7 female), *n* = 4 from the WT group (2 male/2 female), n = 6 from the KspCre^+/−^Nfat5^fl/fl^ group (4 male/2 female), and n = 4 from the Nfat5^fl/fl^ group (2 male/2 female). For further analysis, the 19 mice from the Aqp2‐Cre^+/−^, Nfat5^fl/fl^ and WT groups were combined and referred to as the Ctr control group, except in cases where Ctr was defined separately. There were no further inclusion or exclusion criteria for either test group. All mice were kept in individually ventilated cages (IVCs) at a temperature of 20°C to 24°C, humidity of 45% to 60%, and 15 air exchanges per hour. Feeding (1314 breeding diet from Altromin, Lage, Germany) and drinking ad libitum. The animal experiments in this study were approved by the Commission for Laboratory Animal Husbandry of the State of Saxony‐Anhalt, Germany, and conducted according to local guidelines (approval code: 203.h‐42502‐2‐1250 MLU).

### 2.3 Preparation of samples for next‐generation sequencing

Total RNA from renal cortex and renal inner medulla was isolated with the Gen Elute Mammalian Total RNA prep kit (RTN350‐1KT, Sigma‐Aldrich, St. Louis, MO, USA) according to the manufacturer's instructions and subjected to next‐generation sequencing. Quality control, sequencing and bioinformatics were performed by Novogene Company Limited (Cambridge, United Kingdom) as a service and as described before.^16^

### 2.4 RNA quantification and qualification

RNA degradation and contamination were monitored on 1% agarose gels. RNA purity was checked using the NanoPhotometer® spectrophotometer (Implen GmbH, Munich, Germany). RNA integrity and quantitation were assessed using the RNA Nano 6000 Assay Kit of the Bioanalyzer 2100 system (Agilent Technologies, Santa Clara, CA, USA; RRID:SCR_019715).

### 2.5 Library preparation for next‐generation sequencing

A total amount of 1 μg RNA per sample was used as input material for the RNA sample preparations. Sequencing libraries were generated using NEBNext® UltraTM RNA Library Prep Kit for Illumina® (New England Biolabs, Ipswich, MA, USA) following manufacturer's recommendations and index codes were added to attribute sequences to each sample. Briefly, mRNA was purified from total RNA using poly‐T oligo‐attached magnetic beads. Fragmentation was carried out using divalent cations under elevated temperature in NEBNext First Strand Synthesis Reaction Buffer (5X). First strand cDNA was synthesized using random hexamer primer and M‐MuLV Reverse Transcriptase (RNase H‐). Second‐strand cDNA synthesis was subsequently performed using DNA polymerase I and RNase H. Remaining overhangs were converted into blunt ends via exonuclease/polymerase activities. After adenylation of 3′ ends of DNA fragments, NEBNext Adaptors with hairpin loop structures were ligated to prepare for hybridization. In order to select cDNA fragments of preferentially 150–200 bp in length, the library fragments were purified with the AMPure XP system (Beckman Coulter, Indianapolis, IN, USA). Then 3 μL USER Enzyme (New England Biolabs, Ipswich, MA, USA) was used with size‐selected, adaptor‐ligated cDNA at 37°C for 15 min followed by 5 min at 95°C before PCR. Then PCR was performed with Phusion High‐Fidelity DNA polymerase, universal PCR primers, and index (X) primer. At last, PCR products were purified (AMPure XP system), and library quality was assessed on the Agilent Bioanalyzer 2100 system (Agilent Technologies, Santa Clara, CA, USA; RRID:SCR_019715).

### 2.6 Clustering and sequencing

The clustering of the index‐coded samples was performed on a cBot Cluster Generation System using PE Cluster Kit cBot‐HS (Illumina, San Diego, CA, USA) according to the manufacturer's instructions. After cluster generation, the library preparations were sequenced on an Illumina NovaSeq 6000 platform, and paired‐end reads were generated.

### 2.7 Data analysis and quality control

Clean reads were obtained from raw reads of FASTQ format by removing reads containing adapter and poly‐N sequences and reads with low quality. The Q20, Q30, and GC content of the clean data were calculated. All the downstream analyses were based on the clean reads with high quality.^22^, ^23^, ^24^

### 2.8 Mapping to reference genome

Reference genome (ensembl_mus_musculus_grcm38_p6_gca_000001635_8) and gene model annotation files were downloaded from the genome website browser (NCBI/UCSC/Ensembl). Paired‐end clean reads were mapped to the reference genome using HISAT2 (RRID:SCR_015530) software.^25^, ^26^, ^27^

### 2.9 Quantification

HTSeq (RRID:SCR_011867) was used to count the read numbers mapped to each gene, including known and novel genes. The FPKMs (fragments per kilobase per Million mapped fragments) of each gene were calculated based on the length of the gene and reads count mapped to this gene.^28^, ^29^

### 2.10 Differential expression analysis

Differential expression analysis was performed using the DESeq2 R package (RRID:SCR_015687). The resulting *p*‐values were adjusted using the Benjamini and Hochberg's approach for controlling the False Discovery Rate (FDR). Genes with an adjusted *p*‐value <.05 were assigned as differentially expressed.^30^

### 2.11 Functional enrichment analysis

Gene Ontology (GO) annotates genes to biological processes, molecular functions, and cellular components, and Kyoto Encyclopedia of Genes and Genomes (KEGG) annotates genes to pathways. KOBAS software (RRID:SCR_006350) was used to test the statistical enrichment of differential expression genes in KEGG pathways.^31^

### 2.12 Histological analysis

Formalin‐fixed paraffin‐embedded kidney samples were cut into thin sections measuring 1.5 μm (immunofluorescence staining) or 4 μm (Sirius Red staining). These sections were then subjected to a 16‐h incubation at 60°C to remove excess paraffin. Deparaffinization was accomplished using xylene, followed by rehydration through a series of decreasing ethanol solutions and *Aqua bidest*. The stained sections were observed using an Eclipse Ti2‐A microscope and a DS‐Ri2 camera, controlled by the NIS‐Elements software (Nikon Inc., Melville, NY, USA; RRID:SCR_014329).

### 2.13 Immunofluorescence staining

For immunofluorescence (IF) staining, rehydrated tissue sections underwent a 12‐min pressure‐cooking process in 1x Target Retrieval Solution (Cat. #S1699, Agilent Technologies, Inc., Santa Clara, CA, USA). To minimize nonspecific binding, proteins were blocked for 1 h at room temperature using 5% skimmed milk in TBS‐T. Primary antibodies, diluted in Antibody‐Diluent (Cat. #S3022, Agilent Technologies, Inc., Santa Clara, CA, USA), were then incubated overnight at 4°C. The appropriate secondary antibody was applied for 1 h at room temperature. Sections were mounted using Immu‐MountTM (Cat. #9990402, Thermo Fisher Scientific Inc., Waltham, MA, USA). The following primary antibodies were used: Aquaporin‐2 (polyclonal rat; #20102rs; BiCell Scientific, Maryland Heights, MO, USA; RRID:AB_2910118), Calbindin (monoclonal mouse; #C9848; Sigma‐Aldrich, St. Louis, MO, USA; RRID:AB_476894), CD3 (monoclonal rabbit; #ab16669; abcam, Cambridge, UK; RRID:AB_443425), Cre (monoclonal rabbit; #59238; Cell Signaling Technology, Danvers, MA, USA; RRID:AB_2799562), F4/80 (monoclonal rat; #MCA497GA; Bio‐Rad Laboratories, Hercules, CA, USA; RRID:AB_323806), KIM‐1 (polyclonal goat; #AF1817; R and D Systems, Minneapolis, MN, USA; RRID:AB_2116446), LY‐6G (polyclonal rat; #127601; BioLegend, San Diego, CA, USA; RRID:AB_1089180), Megalin (monoclonal mouse; #ab184676; abcam, Cambridge, UK; RRID:AB_2910117), NCC (polyclonal rabbit; #AB3553; Merck Millipore, Burlington, MA, USA; RRID:AB_571116), NGAL (polyclonal goat; #AF1857; R and D Systems, Minneapolis, MN, USA; RRID:AB_355022), NKCC2 (polyclonal rabbit; #LS‐C313275; Lifespan Biosciences, Shirley, MA, USA; RRID:AB_2910114), NKCC2 (polyclonal goat; #ab240542; abcam, Cambridge, UK; RRID:AB_2910116) and Vimentin (monoclonal rabbit; #ab92547; abcam, Cambridge, UK; RRID:AB_10562134). The following secondary antibodies were used: anti‐goat Alexa Fluor 488 (donkey; #705‐545‐147; Jackson Immuno Research Labs, West Grove, PA, USA; RRID:AB_2336933), anti‐goat Alexa Fluor 594 (donkey; #705‐585‐147; Jackson Immuno Research Lab, West Grove, PA, USA; RRID:AB_2340433), anti‐mouse Cy3 (donkey; #715‐165‐150; Jackson Immuno Research Labs, West Grove, PA, USA; RRID:AB_2340813), anti‐rabbit Alexa Fluor 488 (donkey; #711‐545‐152; Jackson Immuno Research Labs, West Grove, PA, USA; RRID:AB_2313584), anti‐rabbit Cy3 (goat; #111‐165‐003; Jackson Immuno Research Labs, West Grove, PA, USA; RRID:AB_2338000) and anti‐rat Cy3 (donkey; #712‐165‐150; Jackson Immuno Research Labs, West Grove, PA, USA; RRID:AB_2340666).

### 2.14 Sirius Red staining

Rehydrated tissue sections were stained with the Sirius Red staining kit (Cat. #13425.00250, Morphisto GmbH, Offenbach, Germany) according to the manufacturer's protocol. Stained slices were dehydrated and mounted with Roti®Histokitt II (Cat. #T160.1, Carl Roth GmbH, Karlsruhe, Germany).

### 2.15 Statistics

Differential expression analysis and volcano plot visualization were performed using the DESeq2 R package (RRID:SCR_01568). Dot plot visualizations were generated with GraphPad Prism 8.0 (Dotmatics, Boston, MA, USA; RRID:SCR_002798). Data are presented as means ± *SD*. The *p*‐values were calculated using DESeq2, with values less than .05 considered statistically significant. Heatmap visualization of statistically differentially expressed genes and *z*‐score calculations were conducted using Qlucore Omics Explorer 3.9 (Qlucore, New York, NY, USA). For polar plots, the mean gene expression was calculated per group for selected gene sets (*p*‐value <.05). The expression in control lines was set to 100%, and relative expression changes were calculated as a percentage. Plots were created with the R function *radarchart*.

## 3 RESULTS

### 3.1 Loss of NFAT5 is associated with massive changes in gene expression

We used Nfat5^fl/fl^ mice and bred them either with Ksp1.3‐Cre mice (KspCre^+/−^Nfat5^fl/fl^), to generate distal tubule and CD‐specific deficiency, or with Aqp2‐Cre mice to generate CD PC cell‐specific *Nfat5* (Aqp2Cre^+/−^Nfat5^fl/fl^) deficient mice. Detection of the Cre‐recombinase in different kidney sections served to prove its expression pattern (Figure S1). In KspCre^+/−^Nfat5^fl/fl^ mice, Cre co‐localized with the tubule markers Calbindin (in the DCT and CNT) and Aquaporin‐2 (in the CD). As expected, Aqp2Cre^+/−^Nfat5^fl/fl^ mice exhibited Cre expression exclusively in the collecting duct (Figure S1).

**FIGURE 1:**
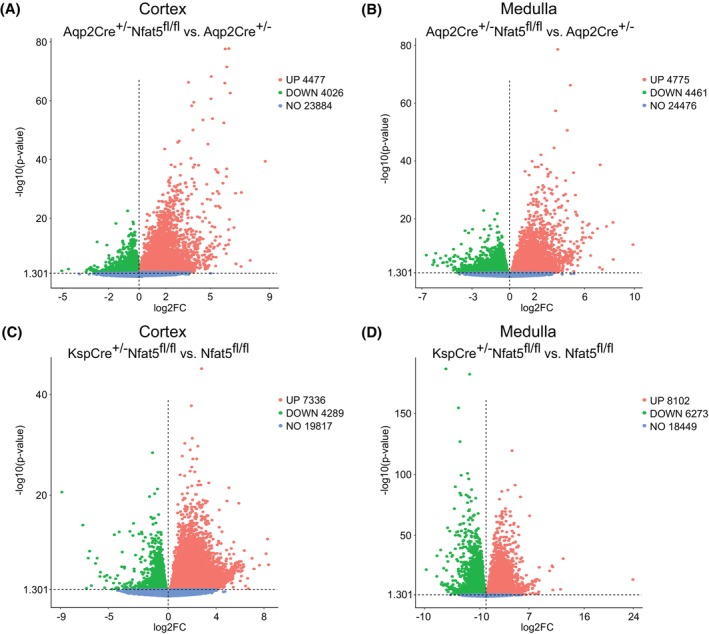
Impact of NFAT5 loss on gene expression patterns in the cortex and inner medulla. (A–D) Volcano Plots showing the upregulated genes (red dots, log2FC >0, *p*‐value <.05) and downregulated genes (green dots, log2FC <0, *p*‐value <.05) between CD‐specific *Nfat5*‐KO kidney Aqp2Cre^+/−^Nfat5^fl/fl^ versus control in the (A) cortex and (B) inner medulla, and between the distal‐tubule‐specific *Nfat5*‐KO kidney KspCre^+/−^Nfat5^fl/fl^ versus control in the (C) cortex and (D) inner medulla. Blue‐dotted genes are not differentially expressed. *p*‐values were calculated with DESeq2.

The analysis of differentially expressed genes in renal cortex (CTX) and inner medulla (IM) showed massive changes in expression levels of both *Nfat5*‐KO models. In Aqp2Cre^+/−^Nfat5^fl/fl^ mice, 8503 genes in the CTX (Figure 1A) and 9236 genes in the IM (Figure 1B) showed an alteration in expression level. The number of differentially expressed genes is higher in KspCre^+/−^Nfat5^fl/fl^ mice. Here, 11 625 genes are differentially expressed in the CTX (Figure 1C) and 14 375 genes in the IM compared to control kidneys (Figure 1D).

### 3.2 Differences in gene expression between KspCre ^+/−^Nfat5^fl/fl^ and Aqp2Cre ^+/−^Nfat5^fl/fl^ kidneys

**FIGURE 2:**
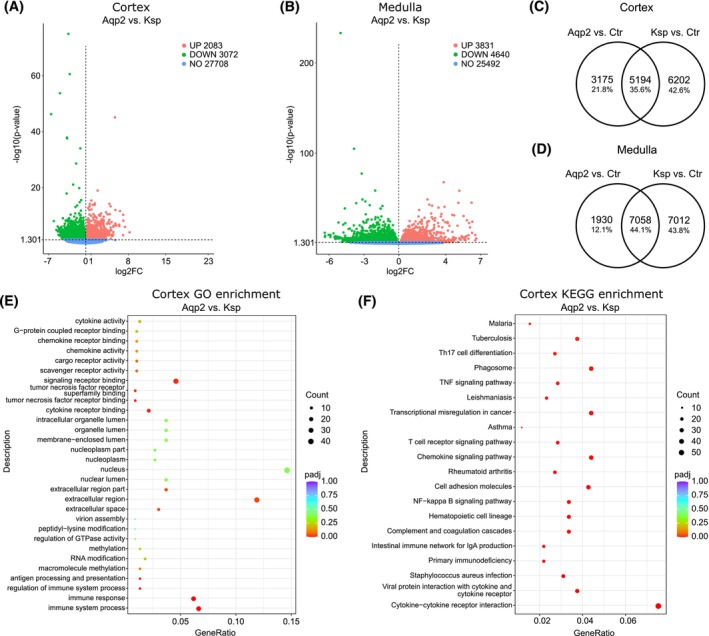
Differences in gene expression between KspCre^+/−^Nfat5^fl/fl^ and Aqp2Cre^+/−^Nfat5^fl/fl^ kidneys in the cortex and inner medulla. (A, B) Volcano plots showing the upregulated genes (red dots, log2FC >0, *p*‐value <.05) and downregulated genes (green dots, log2FC <0, *p*‐value <.05) between CD‐specific *Nfat5*‐KO kidney Aqp2Cre^+/−^Nfat5^fl/fl^ (Aqp2) and the distal‐tubule‐specific *Nfat5*‐KO kidney KspCre^+/−^Nfat5^fl/fl^ (Ksp) in the (A) cortex and (B) inner medulla. Blue dots represent genes that are not differentially expressed. *p*‐values were calculated with DESeq2. (C, D) Venn diagrams illustrating the number of commonly and exclusively differentially expressed genes between Aqp2 versus Ctr (control Aqp2Cre^*+/−*^) and Ksp versus Ctr (control Nfat5^fl/fl^) in the (C) cortex and in the (D) inner medulla. (E, F) Dot Plots presenting the top statistically enriched (E) GO terms and (F) KEGG terms associated with upregulated genes in the cortex between Aqp2 versus Ksp.

We next analyzed differences in gene expression level between kidneys of KspCre^+/−^Nfat5^fl/fl^ and Aqp2Cre^+/−^Nfat5^fl/fl^ mice. Notably, 5155 genes were differentially expressed in the CTX (2083 upregulated, 3072 downregulated) and 8471 genes in the IM (Figure 2A,B) when comparing the *Aqp2*‐KO versus *Ksp*‐KO. 5194 genes were commonly deregulated in both genotypes. An unexpectedly large number of genes were regulated in the CTX of either the Aqp2Cre^+/−^Nfat5^fl/fl^ (3175 genes) or in the KspCre^+/−^Nfat5^fl/fl^ (6202 genes) (Figure 2C). Similar differences were observed in the IM (Figure 2D). The complete lists of differentially expressed genes in *Aqp2*‐KO and *Ksp*‐KO and the list of common and uniquely differentially expressed genes is available in the Supplement Excel File S1.

We used the list of differentially expressed genes between both models for functional analysis using gene ontology terms and KEGG signaling pathways. In kidneys of Aqp2Cre^+/−^Nfat5^fl/fl^ mice, the results showed enriched expression of genes that are associated with GO terms like “*immune response*,” “*cytokine activity*,” or “*TNF receptor binding*,” which was confirmed by KEGG pathway analysis (Figure 2E,F). The results of the functional analysis for downregulated genes are provided in the Figure S2.

**FIGURE 3:**
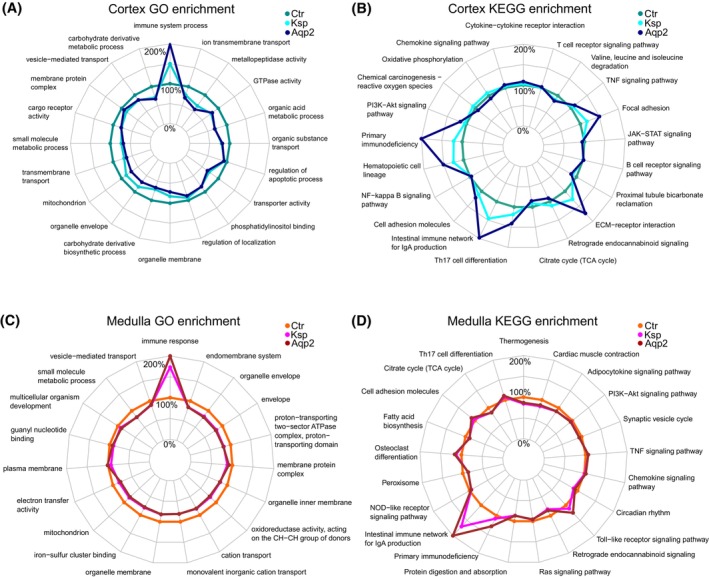
Enrichment analysis reveals increased expression of immune response genes in Aqp2Cre^+/−^Nfat5^fl/fl^ kidneys compared to KspCre^+/−^Nfat5^fl/fl^ kidneys. Polar plots showing the mean expression levels of genes that have been normalized to the control (100%) and are associated with (A, C) enriched GO terms and (B, D) enriched KEGG terms in control (Ctr), Aqp2Cre^+/−^Nfat5^fl/fl^ (Aqp2) and KspCre^+/−^Nfat5^fl/fl^ (Ksp) kidneys in the cortex (A, B) and inner medulla (C, D).

The polar plots show the mean expression level of all genes associated with enriched GO terms in the CTX of control, Aqp2Cre^+/−^Nfat5^fl/fl^ and KspCre^+/−^Nfat5^fl/fl^ mice. Again, the GO term “*immune system process*” showed higher expression levels in the Aqp2Cre^+/−^Nfat5^fl/fl^ mice compared to KspCre^+/−^Nfat5^fl/fl^ mice (Figure 3A), which is also evident in the IM (Figure 3C). Similar differences were observed for enriched KEGG signaling pathways (Figure 3B,D). The analyses show that loss of NFAT5 affected the expression of inflammatory genes in the kidney CTX and IM similarly. Of note, based on the expression level, the response seems to be higher in the Aqp2Cre^+/−^Nfat5^fl/fl^ kidney compared to KspCre^+/−^Nfat5^fl/fl^ kidneys.

### 3.3 Loss of NFAT5 is associated with renal injury and fibrosis

**FIGURE 4:**
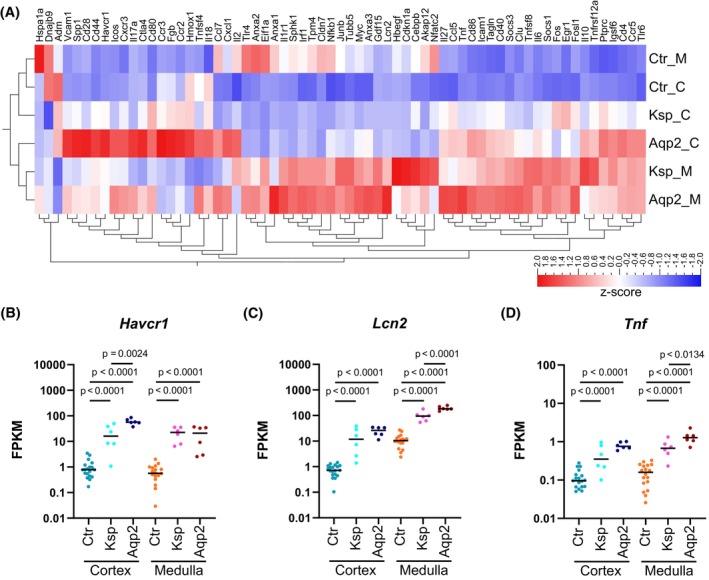
Loss of NFAT5 is associated with kidney injury. (A) Clustered heatmap with the expression *z*‐scores of kidney injury marker genes in the cortex (_C) and in the inner medulla (_M) of control (Ctr), KspCre^+/−^Nfat5^fl/fl^ (Ksp), and Aqp2Cre^+/−^Nfat5^fl/fl^ (Aqp2) kidneys. (B–D) Log‐transformed FPKM (Fragments Per Kilobase per Million mapped fragments) expression levels of three kidney injury marker genes (B) *Havcr1* (hepatitis A virus cellular receptor 1), (C) *Lcn2* (lipocalin 2), and (D) *Tnf* (tumor necrosis factor) in the cortex and inner medulla of control (Ctr), KspCre^+/−^Nfat5^fl/fl^ (Ksp), and Aqp2Cre^+/−^Nfat5^fl/fl^ (Aqp2) kidneys. The dots represent individual samples. *p*‐values were calculated with DESeq2.

The loss of NFAT5 was associated with increased expression levels of several kidney injury markers. The heatmap in Figure 4A shows the expression level of selected genes that are associated with kidney injury. For the majority of the genes, we observed a higher expression level in the *Nfat5*‐KO kidneys. Interestingly, Aqp2Cre^+/−^Nfat5^fl/fl^ compared to KspCre^+/−^Nfat5^fl/fl^ kidneys showed higher expression levels for most of the transcripts. This includes factors like kidney injury molecule 1 (KIM‐1 alias *Havcr1*), lipocalin‐2 (NGAL alias *Lcn2*) or tumor necrosis factor (*Tnf*) (Figure 4A–D).

**FIGURE 5:**
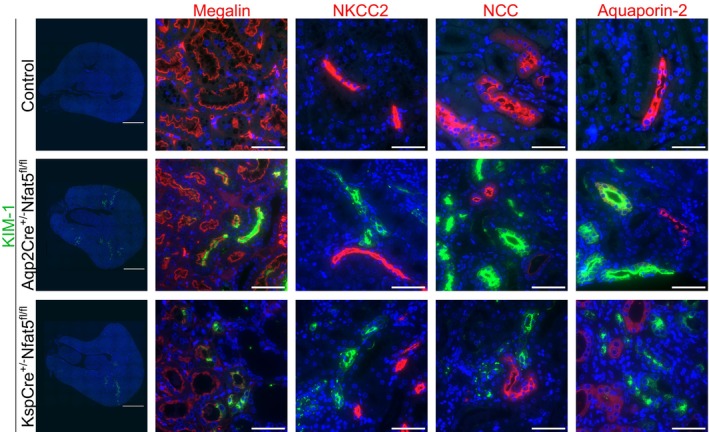
Nephron localization of KIM‐1 in different *Nfat5*‐KO models. Immunofluorescence staining of KIM‐1 (green) with the nephron segment markers (red) Megalin (PT), NKCC2 (TAL), Calbindin (DCT and CNT), and Aquaporin‐2 (CD). In both *Nfat5*‐KO models, KIM‐1 expression was restricted to the PT. Scale bars: 1000 μm and 50 μm.

To identify the nephron segments that are affected by the loss of NFAT5, we used different segment‐specific markers for co‐staining with the kidney injury markers KIM‐1 and NGAL. In contrast to control kidneys, we observed KIM‐1 positivity along with megalin in NFAT5‐deficient kidneys, indicative of proximal tubule injury (Figure 5). NGAL‐positive areas were localized in the entire nephron (Figure S3).

**FIGURE 6:**
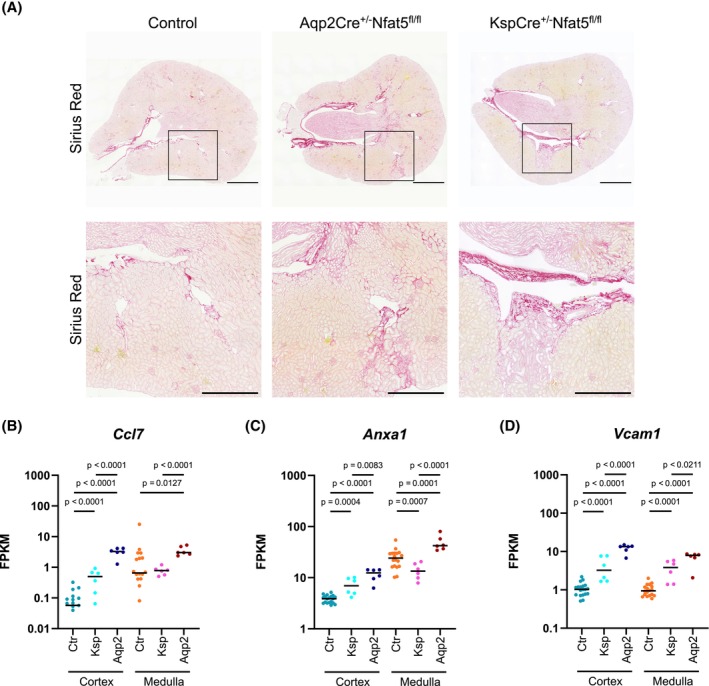
Loss of NFAT5 is associated with renal fibrosis. (A) Picro Sirius Red staining of *Nfat5*‐KO and control kidneys. Scale Bar: 1000 μm (upper row) and 500 μm (lower row). (B–D) Log‐transformed FPKM (Fragments Per Kilobase per Million mapped fragments) expression levels of three renal fibrosis marker genes (B) *Ccl7* (C‐C motif chemokine ligand 7), (C) *Anxa1* (annexin A1), and (D) *Vcam1* (vascular cell adhesion molecule 1) in the cortex and inner medulla of control (Ctr), KspCre^+/−^Nfat5^fl/fl^ (Ksp), and Aqp2Cre^+/−^Nfat5^fl/fl^ (Aqp2) kidneys. The dots represent individual samples. *p*‐values were calculated with DESeq2.

Furthermore, Sirius red staining of kidneys of both *Nfat5*‐KO models showed large fibrotic areas (Figure 6A), and were also present in the CTX. Accordingly, genes associated with fibrosis (*Ccl7*, *Anxa1*, and *Vcam1*) showed significantly higher expression levels in Aqp2Cre^+/−^Nfat5^fl/fl^ mice and KspCre^+/−^Nfat5^fl/fl^ mice (Figure 6B–D).

**FIGURE 7:**
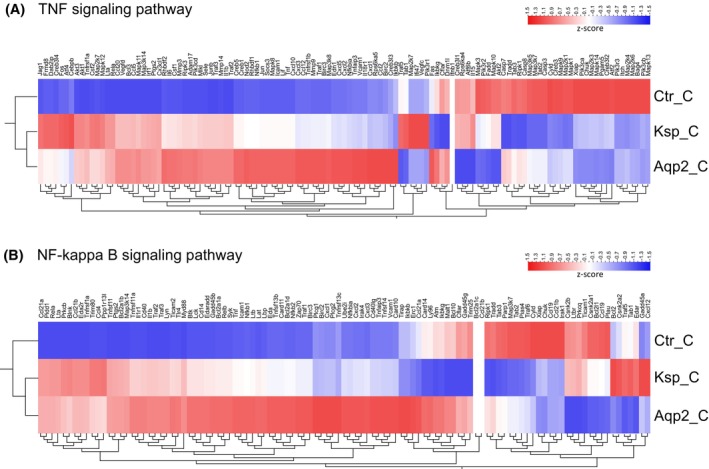
Loss of NFAT5 is associated with increased expression of TNF signaling and NF‐kappa B signaling pathway genes in the cortex of Aqp2Cre^+/−^Nfat5^fl/fl^ kidneys. Clustered heatmaps with the expression *z*‐scores of (A) TNF signaling pathway genes and (B) NF‐kappa B signaling pathway genes in the cortex (_C) of control (Ctr), KspCre^+/−^Nfat5^fl/fl^ (Ksp), and Aqp2Cre^+/−^Nfat5^fl/fl^ (Aqp2) kidneys.

**FIGURE 8:**
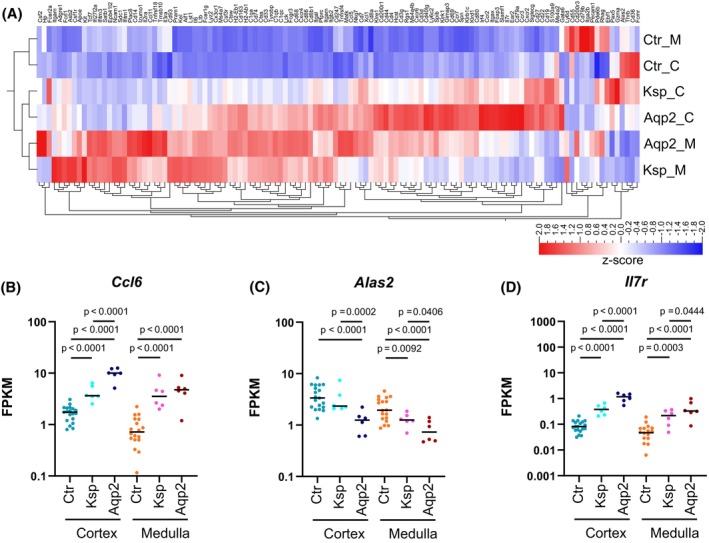
Loss of NFAT5 is associated with the activation of the immune system. (A) Clustered heatmap with the expression *z*‐scores of immune cell‐specific marker genes in the cortex (_C) and in the inner medulla (_M) of control (Ctr), KspCre^+/−^Nfat5^fl/fl^ (Ksp), and Aqp2Cre^+/−^Nfat5^fl/fl^ (Aqp2) kidneys. (B–D) Log‐transformed FPKM (Fragments Per Kilobase per Million mapped fragments) expression levels of the immune cell‐specific marker genes (B) *Ccl6* (C‐C motif chemokine ligand 6), (C) *Alas2* (aminolevulinic acid synthase 2, erythroid), and (D) *Il7r* (interleukin 7 receptor) in the cortex and inner medulla of control (Ctr), KspCre^+/−^Nfat5^fl/fl^ (Ksp), and Aqp2Cre^+/−^Nfat5^fl/fl^ (Aqp2) kidneys. The dots represent individual samples. *p*‐values were calculated with DESeq2.

**FIGURE 9:**
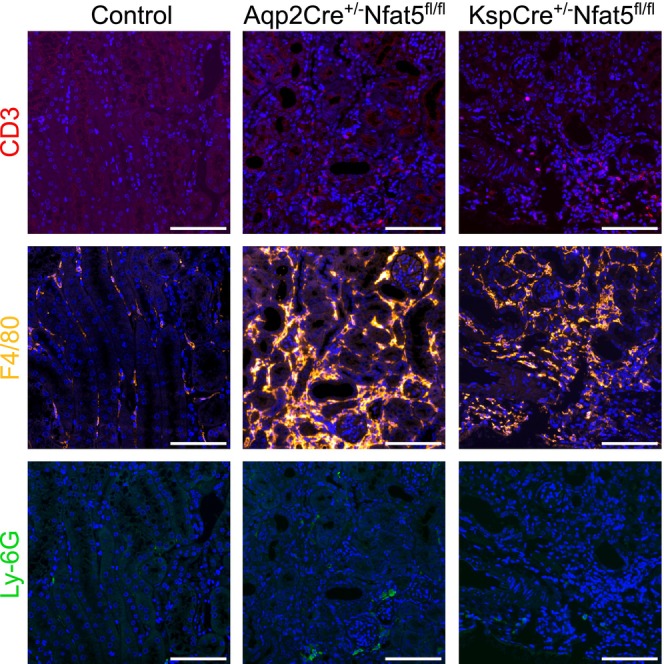
Immune cell infiltration following *Nfat5* knockout in the kidney. Immunofluorescence staining of immune cell markers CD3 (T‐lymphocytes), F4/80 (macrophages), and Ly‐6G (granulocytes) on *Nfat5*‐KO and control kidneys. Following *Nfat5*‐KO kidneys showed areas with infiltrating immune cells. Scale bar: 100 μm.

Since fibrosis results from inflammatory reactions, we also focused on the effects of NFAT5 deletion on pro‐inflammatory‐related pathways. In both *Nfat5*‐KO models, for example, TNF and NF‐kappa B pathway‐related genes were highly expressed in the CTX (Figure 7A,B) and IM (Figure S4A,B), again, with higher gene expression rates in the Aqp2Cre^+/−^Nfat5^fl/fl^ mice. This is also highlighted by the heatmap shown in Figure 8A. The expression of further immune response genes was higher in the Aqp2Cre^+/−^Nfat5^fl/fl^ mice compared to KspCre^+/−^Nfat5^fl/fl^ kidneys (Figure 8A–D). To distinguish between different immune cells, we stained immune cell markers like CD3 for T‐lymphocytes, F4/80 for macrophages, and Ly‐6G for granulocytes. Our data confirmed infiltration of *Nfat5*‐KO kidneys with immune cells (Figure 9) and indicated that F4/80‐positive macrophages might contribute to the observed inflammatory response since macrophages are localized within the fibrotic areas of both knockout models (Figure S5).

These data indicate that the loss of NFAT5 leads to kidney injury, which drives or is driven by a pro‐inflammatory immune response and culminates in fibrosis. Both knockout models also show injured nephron segments that are not affected by the knockout.

## 4 DISCUSSION

Our study provides two major findings: First, *Nfat5* deficiency alone in the distal nephron is sufficient to cause severe kidney fibrosis that represents the common pathological feature and signs of chronic kidney disease (CKD). Mechanistically, *Nfat5*‐KO‐mediated kidney injury seemed to be mainly attributed to an activation of pro‐inflammatory signaling and immune cell infiltration. Second, kidney injury was also observed upstream of *Nfat5* depletion, e.g., in proximal tubules, suggesting a functional feedback mechanism that transfers deleterious signals from distal to proximal segments of the nephron. The comparison of two different *Nfat5*‐KO models, which either affect the collecting duct only or the entire distal nephron, indicates that NFAT5 fulfills a hitherto unexpected cortical function irrespective of adaptation to hypertonicity.

The primary function of the kidney is to excrete waste through urine while conserving water through reabsorption. Since water moves passively, high concentrations of sodium, chloride, and urea in the kidney medulla create the necessary driving force. This high osmolarity forces cells to produce osmo‐protective substances, like sorbitol via the polyol pathway, to prevent shrinking. Cells adapt to hypertonicity through NFAT5, the key transcription factor for regulating hypertonicity‐induced gene expression.^32^, ^33^ Moreover, several studies showed an involvement of NFAT5 in renal injury. Some studies suggest that NFAT5 provides protection against acute kidney injury,^34^, ^35^ while others indicate the opposite effect.^36^, ^37^ These observations indicate that NFAT5 may have functions beyond adapting to hypertonicity, with its effects varying depending on the specific circumstances studied.

Recently, using a targeted deletion strategy, we generated a PC‐specific *Nfat5*‐KO mouse model using an *Aqp2‐*driven Cre‐deleter (Aqp2Cre^+/−^Nfat5^fl/fl^) that results in a diabetes insipidus‐like phenotype.^17^ In another study, Ono et al. used a tamoxifen‐inducible Pax8‐Cre deleter model to delete NFAT5 in renal tubular cells and identified differentially expressed genes via transcription start site (TSS) analysis.^18^ In both models, a couple of genes like *Prok2*, *Slc14a2*, or *Nr2e3* are commonly downregulated, and *Tacr1*, *Havcr1*, or *Mmp7* are consistently upregulated. To expand our understanding of NFAT5 function and downstream cascades, our gene expression analyses further distinguish between cortex and inner medulla and different tubular segments of the kidney.

A key finding from the two independent *Nfat5* knockout models used in this study is that the loss of NFAT5 is directly associated with a kidney injury‐like phenotype. This is a major difference compared with the findings of Ono et al.^18^, since they observed a severe effect on kidney injury after unilateral ureteral obstruction in NFAT5‐KO mice compared to control and with other studies where experimentally induced kidney injury was found to be more severe in *Nfat5*‐deficient models.^34^, ^35^Despite this, we show that *Nfat5*‐KO in the distal nephron causes strong activation of inflammatory responses and immune cell infiltration. We propose that NFAT5 loss diminishes the ability of medullary cells to resist hypertonicity, leading to cell stress and subsequent immune system activation. Notably, when comparing our data with transcriptomic profiles from various pathophysiological renal conditions,^38^, ^39^ there is a significant overlap in deregulated gene expression patterns. Considering that, our *Nfat5*‐KO mice developed fibrosis, and based on the differences in gene expression patterns, one possibility could be that cytokine‐triggered inflammatory response and immune cell infiltration could contribute to the observed changes.

The second key observation is that the loss of NFAT5 in the distal nephron induces cortical fibrosis and upregulates the kidney injury markers KIM‐1 and NGAL in proximal tubules, even though these regions of the kidney are not exposed to hypertonic stress and have normal NFAT5 expression levels. These observations are supported by analyses of medullary versus cortical alterations of gene expression. These data clearly demonstrate that *Nfat5* knockout affects upstream segments of the tubular system, not prone to the knockout. This suggests a feedback mechanism that functionally links distal and proximal nephron segments. The exact mechanism remains totally unclear at this moment and needs to be addressed in future studies. Notably, Aqp2Cre^+/−^Nfat5^fl/fl^ mice, where the *Nfat5*‐KO is limited to the collecting duct, exhibited higher changes in immune and injury‐related genes than KspCre^+/−^Nfat5^fl/fl^ mice, which lack NFAT5 from the thick ascending limb to the collecting duct. These findings indicate gradual differences in our *Nfat5* knockout models. Therefore, we propose that exocrine signaling factors like cytokine signaling, miRNA or exosomes could contribute to this process, among other potential determinants. However, further studies are needed to clarify their specific roles and mechanisms.

Cytokines can activate the immune system, which in turn can induce kidney injury. NFAT5 has been shown to regulate the expression of TNF (tumor necrosis factor),^40^ a key cytokine in orchestrating inflammatory responses.^41^ Interestingly, the loss of NFAT5 is associated with increased TNF expression, and elevated TNF levels are known to attract immune cells, particularly macrophages, and promote apoptosis.^42^, ^43^ These findings align with our observations that *Tnf* is elevated in both *Nfat5* knockout models. Thus, the immune cell infiltration observed in our *Nfat5* knockout models is likely due to increased TNF expression. To confirm this specific cortical effect on proximal tubules by NFAT5 loss in distal tubules, new experimental setups must be developed for future studies.

In summary, by separately analyzing gene expression in the medullary and cortical regions of the kidney, we found that NFAT5 loss leads to kidney injury beyond just hypertonic areas. The strong correlation between inflammatory response levels, immune cell infiltration, injury severity, and fibrosis extent suggests that cytokines are the primary mediators of stress signals within the kidney. Therefore, NFAT5 plays a crucial role not only in osmotic stress adaptation but also in cytokine signaling.

## AUTHOR CONTRIBUTIONS

The first author positions were determined alphabetically. Kristina Engel analyzed and visualized the next‐generation sequencing data; Vera Anna Kulow performed the histological analysis and staining; Dmitry Chernyakov prepared the next‐generation sequencing samples; Edith Willscher visualized the functional enrichment analyses; Kristina Engel and Vera Anna Kulow designed the figures; Bayram Edemir acquired funding, designed the project, and supervised the project; Michael Fähling and Bayram Edemir took the lead in writing the manuscript. All authors revised and contributed to the final manuscript.

## DISCLOSURES

The authors declare no conflicts of interest.

## Supporting information


Figure S1.

Figure S2.

Figure S3.

Figure S4.

Figure S5.

Data S1.

Text S1.

## Data Availability

The Aqp2Cre^+/−^Nfat5^fl/fl^ and Aqp2‐Cre^+/−^ RNA‐sequencing (RNA‐seq) data were collected in a previous study^16^ and have been deposited in the public repository Gene Expression Omnibus at https://www.ncbi.nlm.nih.gov/geo/query/acc.cgi?acc=GSE195881 under accession number GSE195881. The RNA‐seq data for KspCre^+/−^Nfat5^fl/fl^, Nfat5^fl/fl^ and WT can be found in the same repository under accession number GSE276474. The authors declare that all other data supporting the findings of this study are available in the paper and its supplementary information files. Values for all data points in the graphs can be found in the Supplement Excel File S1.
