# Supplementary figures and images for: Segment specific loss of NFAT5 function in the kidneys is sufficient to induce a global kidney injury like phenotype
Source: FASEB J. 2025 Jan 28;39(2):e70352. doi: 10.1096/fj.202402497R (PMC11774485; doi:10.1096/fj.202402497R)

Supplemental Figure S1

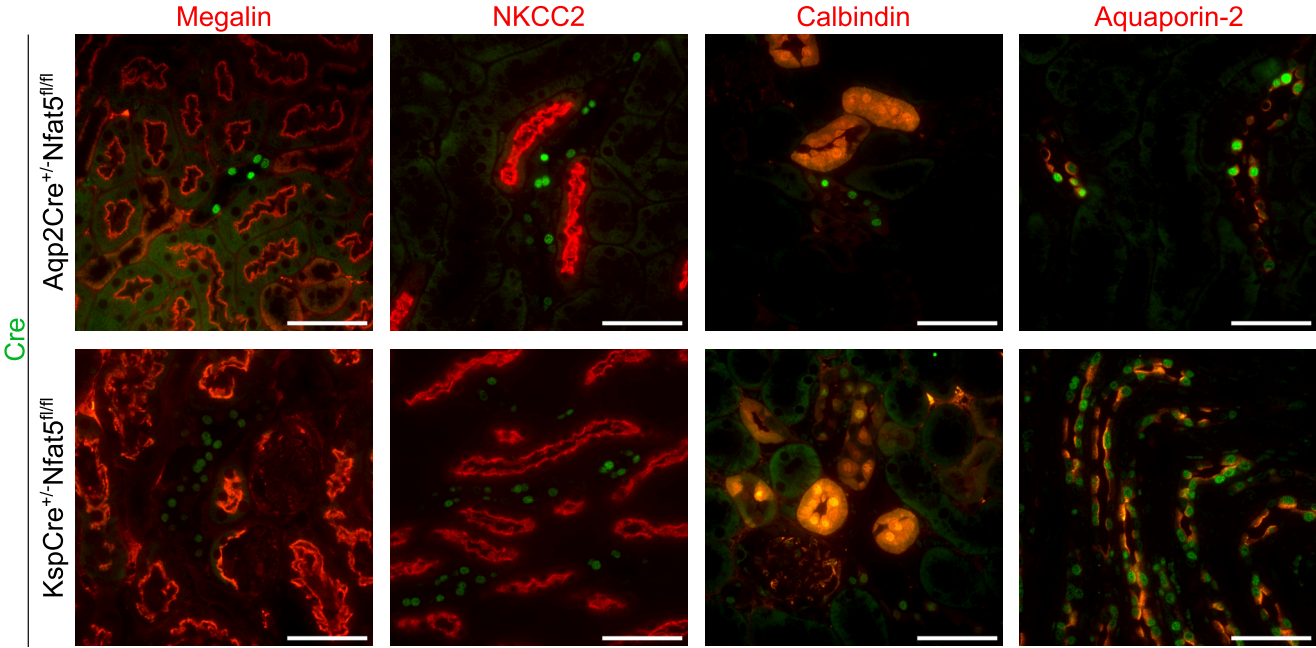

Supplement: Supplementary file 1 — Figure S1. [file FSB2-39-e70352-s002.pdf]

# Supplemental Figure S2

A

## Cortex GO enrichment Aqp2 vs. Ksp

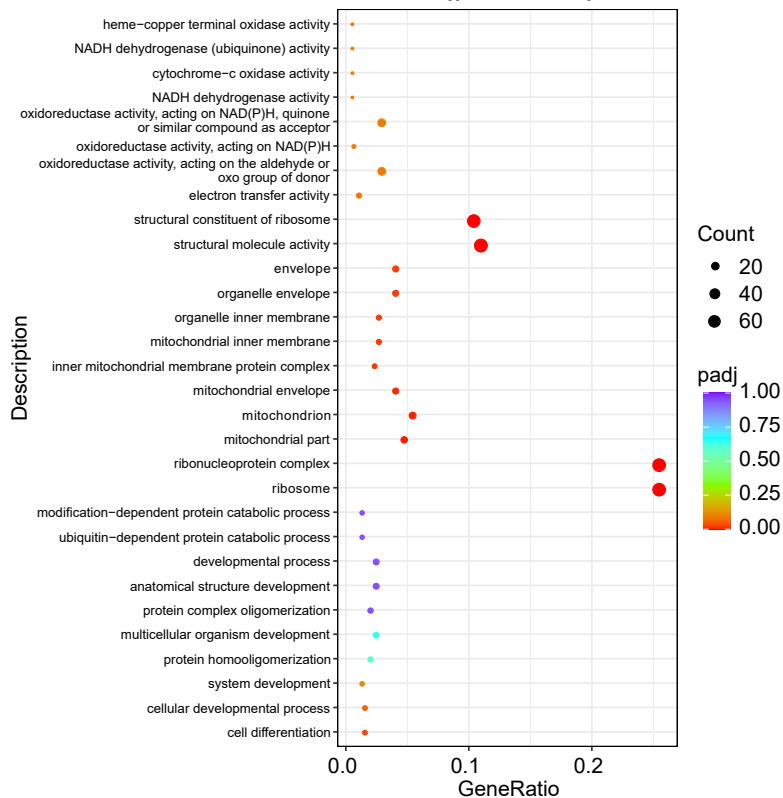

B

## Cortex KEGG enrichment Aqp2 vs. Ksp

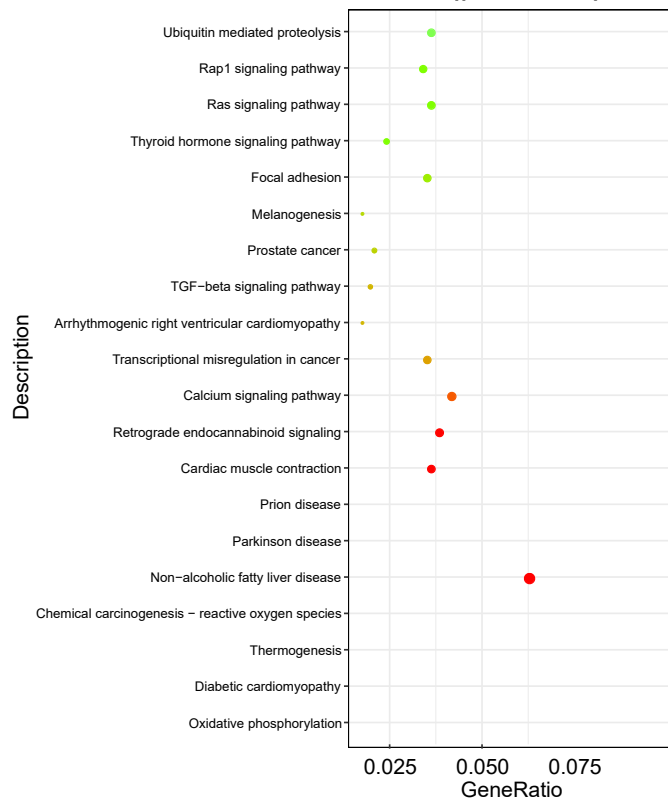

Supplement: Supplementary file 2 — Figure S2. [file FSB2-39-e70352-s006.pdf]

Control

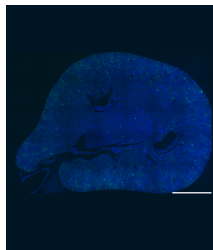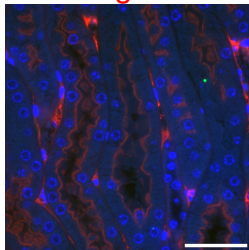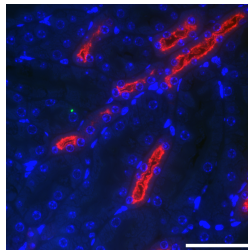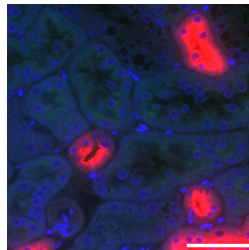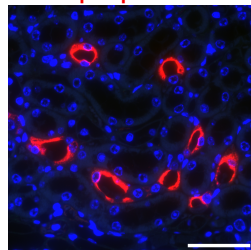Aqp2Cre<sup>+/-</sup>Nfat5<sup>fl/fl</sup>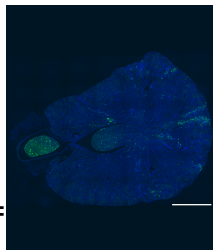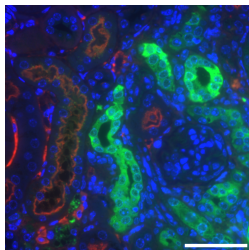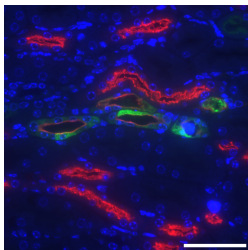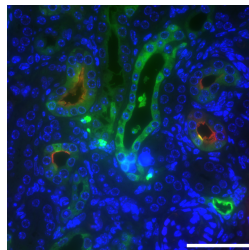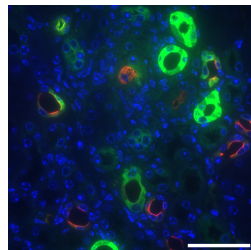KspCre<sup>+/-</sup>Nfat5<sup>fl/fl</sup>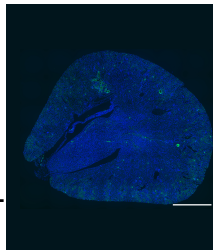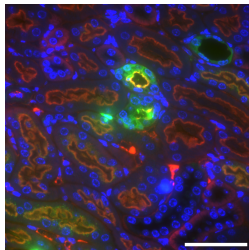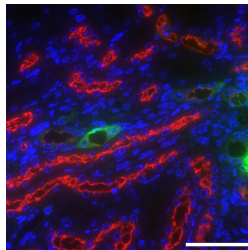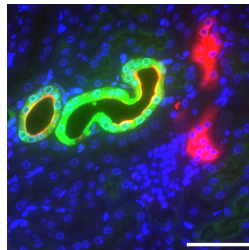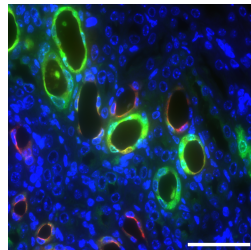

Supplement: Supplementary file 3 — Figure S3. [file FSB2-39-e70352-s003.pdf]

## A TNF signaling pathway

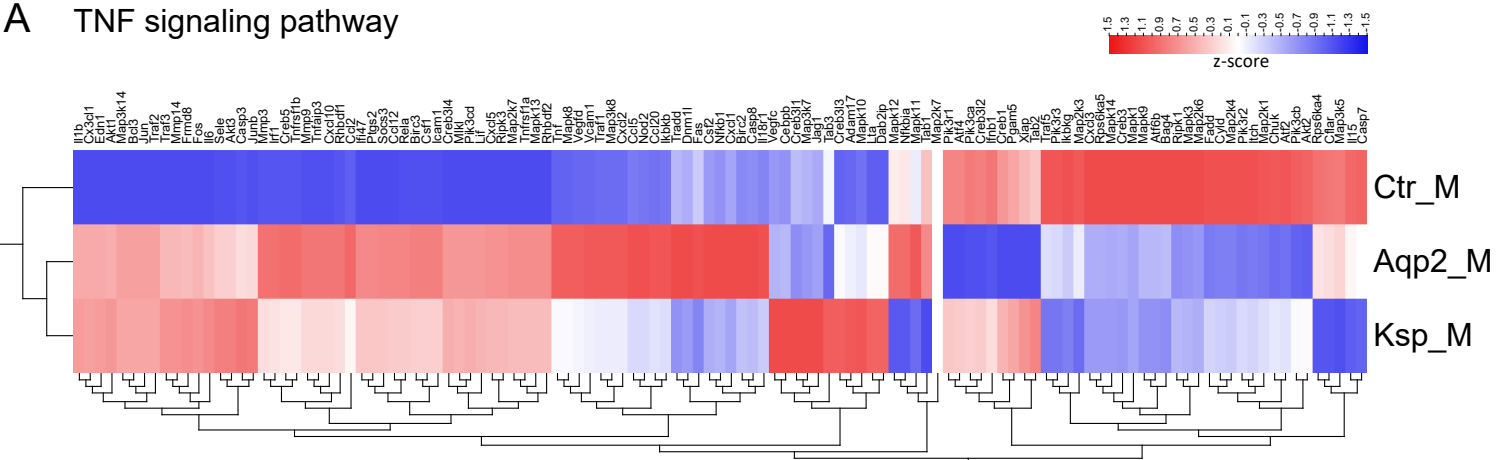

**B** NF-kappa B signaling pathway

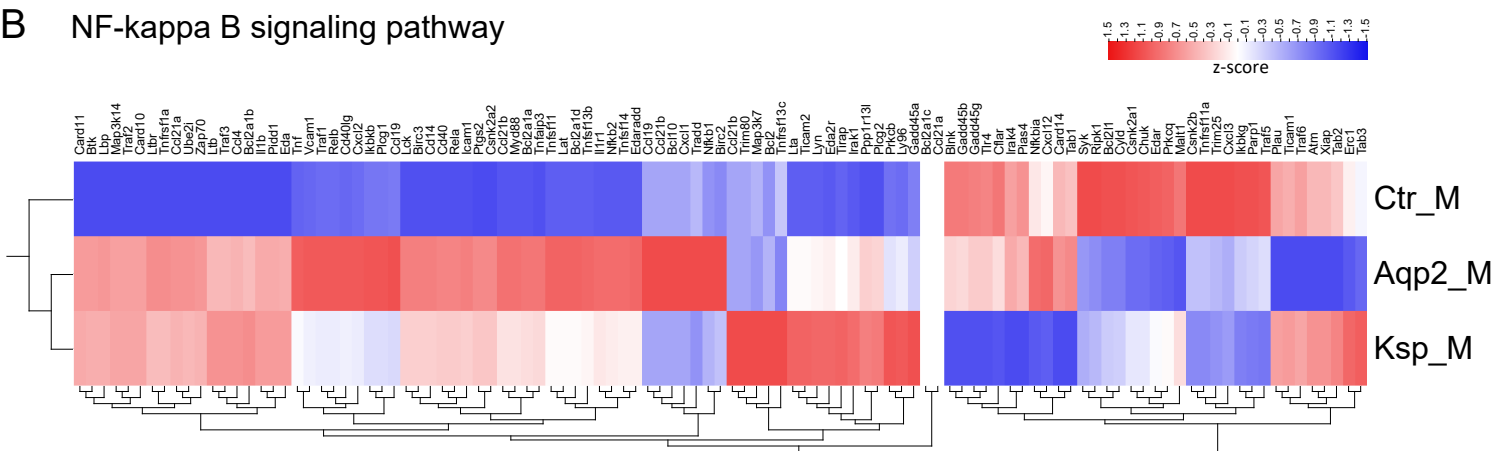

Supplement: Supplementary file 4 — Figure S4. [file FSB2-39-e70352-s001.pdf]

Supplemental Figure S5

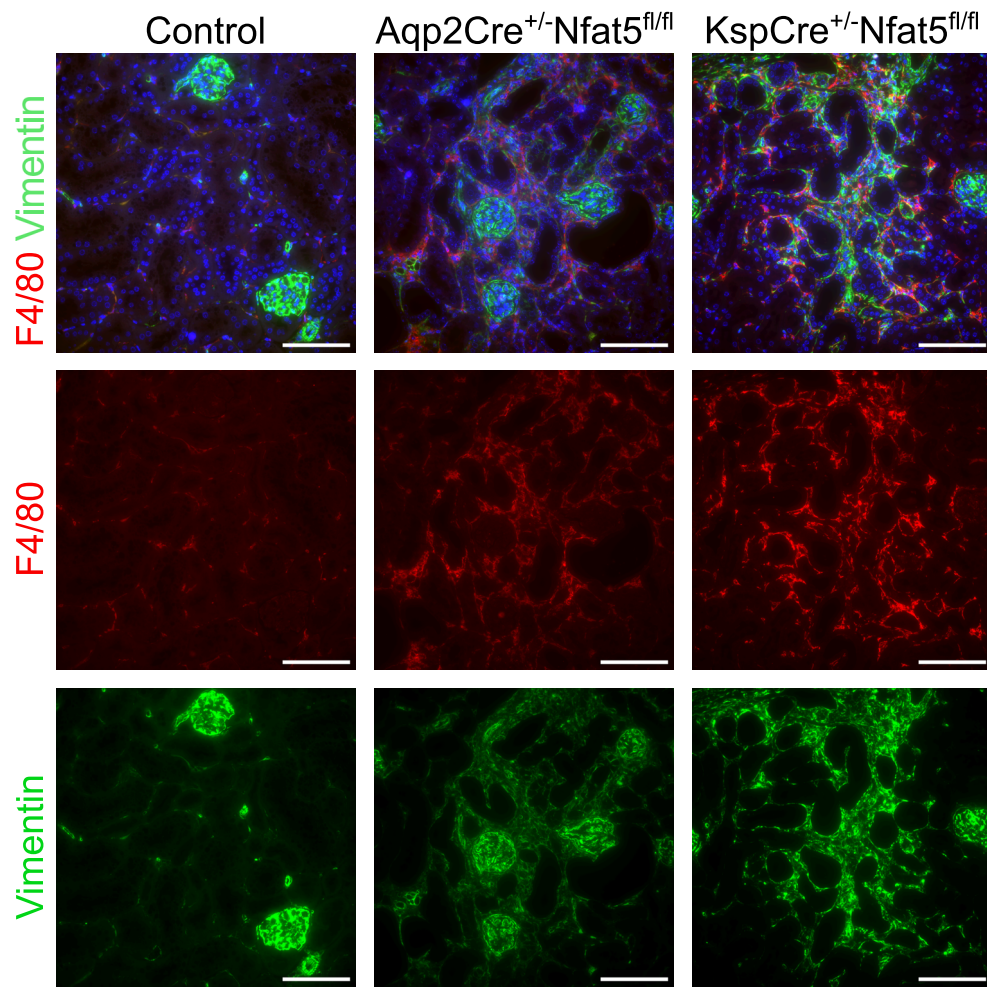

Supplement: Supplementary file 5 — Figure S5. [file FSB2-39-e70352-s005.pdf]
