# Supplementary material for: Segment specific loss of NFAT5 function in the kidneys is sufficient to induce a global kidney injury like phenotype
Source: FASEB J. 2025 Jan 28;39(2):e70352. doi: 10.1096/fj.202402497R (PMC11774485; doi:10.1096/fj.202402497R)
Supplement: Supplementary file 7 — Text S1. [file FSB2-39-e70352-s004.docx]

**Segment specific loss of NFAT5 function in the kidneys is sufficient to induce a global kidney injury like phenotype**

Kristina Engel^1*^, Vera Anna Kulow^2*^, Dmitry Chernyakov^1^, Edith Willscher^1^, Michael Fähling^2^, Bayram Edemir^1, 3^

^1^ Department of Medicine, Hematology and Oncology, Martin Luther University Halle-Wittenberg, Halle (Saale), Germany

^2^ Charité – Universitätsmedizin Berlin, corporate member of Freie Universität Berlin and Humboldt-Universität zu Berlin, Institute of Translational Physiology, Charitéplatz 1, 10117 Berlin, Germany

^3^ Institute for Physiology and Pathophysiology, Zentrum für Biomedizinische Ausbildung und Forschung (ZBAF), Witten/Herdecke University, Witten, Germany

^*^K. Engel und V. A. Kulow should be considered joint first authors.

**Supplement**

Supplement Figure 1: Localization of Cre recombinase in different *Nfat5*-KO models.

Supplement Figure 2: Enrichment analysis between KspCre^+/-^Nfat5^fl/fl^ and Aqp2Cre^+/-^Nfat5^fl/fl^ kidneys in the cortex.

Supplement Figure 3: Nephron localization of NGAL in different *Nfat5*-KO models.

Supplement Figure 4: Loss of NFAT5 is associated with increased expression of TNF signaling and NF-kappa B signaling pathway genes in the inner medulla of Aqp2Cre^+/-^Nfat5^fl/fl^ kidneys.

Supplement Figure 5: Localization of Macrophages in fibrotic areas in different *Nfat5*-KO models.

Supplement Excel File 1: Supporting data file with values for all figures

**Supplement Figure 1: Localization of Cre recombinase in different *Nfat5*-KO models.** Immunofluorescence staining of Cre (green) with the nephron segment markers (red) Megalin (PT), NKCC2 (TAL), Calbindin (DCT and CNT) and Aquaporin-2 (CD). In the Aqp2Cre^+/-^Nfat5^fl/fl^ model Cre was localized exclusively in the CD. In the KspCre^+/-^Nfat5^fl/fl^ model Cre was expressed in the distal part of the nephron including DCT, CNT and CD. Scale bar: 50 µm.

**Supplement Figure 2: Enrichment analysis** **between KspCre^+/-^Nfat5^fl/fl^ and Aqp2Cre^+/-^Nfat5^fl/fl^ kidneys in the cortex.** Dot Plots presenting the top statistically enriched **(A)** GO terms and **(B)** KEGG terms associated with downregulated genes in the cortex between Aqp2 vs. Ksp.

**Supplement Figure 3:** **Nephron** **localization of NGAL in different *Nfat5*-KO models.** Immunofluorescence staining of NGAL (green) with the nephron segment markers (red) Megalin (PT), NKCC2 (TAL), Calbindin (DCT and CNT) and Aquaporin-2 (CD). NGAL was expressed throughout the nephron in both *Nfat5*-KO models. Scale bar: 50.

**Supplement Figure 4: Loss of NFAT5 is associated with increased expression of TNF signaling and NF-kappa B signaling pathway genes in the inner medulla of Aqp2Cre^+/-^Nfat5^fl/fl^ kidneys.** Clustered Heatmaps with the expression z-scores of **(A)** TNF signaling pathway genes and **(B)** NF-kappa B signaling pathway genes in the inner medulla (_M) of control (Ctr), KspCre^+/-^Nfat5^fl/fl^ (Ksp) and Aqp2Cre^+/-^Nfat5^fl/fl^ (Aqp2) kidneys.

**Supplement Figure 5:** **Localization of Macrophages in fibrotic areas in different *Nfat5*-KO models.** Immunofluorescence staining of Vimentin (green) for fibrosis with the macrophage marker F4/80 (red). Co-staining of Vimentin and F4/80 show macrophage infiltration in the fibrotic areas of the kidney following *Nfat5*-KO. Scale bar: 100 µm.
